# Off-Pump Coronary Artery Bypass Grafting Surgery: A Valuable 2-Day Experience

**DOI:** 10.21315/mjms2022.29.6.15

**Published:** 2022-12-22

**Authors:** Nadia Hanom Ishak, Soon Eu Chong, Huda Zainal Abidin, Ahmad Zuhdi Mamat, Ariffin Marzuki Mokthar, Mohd Zamrin Dimon

**Affiliations:** 1 Department of Anaesthesiology and Intensive Care, Faculty of Medicine, Universiti Teknologi MARA, Selangor, Malaysia; 2 Department of Anaesthesiology and Intensive Care, School of Medical Sciences, Universiti Sains Malaysia, Kelantan, Malaysia; 3 Department of Surgery, School of Medical Sciences, Universiti Sains Malaysia, Kelantan, Malaysia; 4 Department of Cardiovascular and Thoracic Surgery, Faculty of Medicine, Universiti Teknologi MARA, Selangor, Malaysia

**Keywords:** off-pump, cardiac aneasthesia, ischaemic heart disesase

## Abstract

Currently, coronary artery disease (CAD) has been identified as the leading cause of mortality in Malaysia and in other countries worldwide. Genetic predisposition and comorbidities such as hypertension and diabetes mellitus, gender, lifestyle, and several other risk factors can contribute to the development of CAD. Pharmacological and surgical treatments play a vital role in improving the quality of life of patients with CAD. New surgical techniques and continuous interventions have been introduced to improve the treatment outcome. Recently, cardiothoracic teams from Universiti Teknologi MARA (UiTM) and Universiti Sains Malaysia (USM) have conducted a 2-day live workshop on off-pump coronary artery bypass (OPCAB) surgery. In this brief communication, we share the highlights and clinical tips of the OPCAB surgery gained from the collaboration.

## Introduction

According to the World Health Organization (WHO), 98.9 deaths per 100,000 population due to coronary artery disease (CAD) have been reported in Malaysia for 2012 (1). As per the National Cardiovascular Disease database, Malaysians commonly develop acute coronary syndrome at a younger age (mean age 55.9–59.1 years old) than those in developed countries (63.4–68.0) (2). There exist a number of national and international guidelines on the management of CAD. Patients are treated accordingly based on the severity of the CAD. Those who are not amenable to medical treatment or coronary angioplasty will be referred to the cardiac surgery team for coronary artery bypass grafting (CABG).

Conventionally, cardiopulmonary bypass (CPB) machine is a fundamental component of CABG, which renders the heart motionless (cardioplegic arrest) and bloodless and, at the same instance, maintains the perfusion to vital organs. Off-pump CABG (OPCAB) surgery was first performed during the 1960s based on the case series described by Professor Kolesov, who is one of the pioneers of cardiovascular surgery (3). However, this novel technique took a hiatus due to the advancement of CPB and cardioplegia. It emerged again in the mid-1980s at centres with limited resources and isolated cases. Subsequently, an extensive series of OPCAB by Buffolo et al. (4) and Benetti et al. (5) were published, and the deemed old technique regained its momentum. Despite being technically challenging for both cardiac surgeons and anaesthesiologists, its potential benefits are closely linked to the avoidance of CPB usage (6).

Upon the initiation of the CPB machine, the blood comes into contact with the extracorporeal circuit of the CPB machine, which then activates the humoral and cellular mediators, leading to a number of adverse consequences related to systemic inflammatory response (7). This response subsequently results in known postoperative pitfalls, namely, coagulopathies, respiratory failure, myocardial infarction and renal dysfunction (8). Post-operative neurological and cognitive dysfunctions are other pertinent issues that need to be addressed. It is believed to be caused by the micro-embolisation of gaseous and particulate matter from lipids and blood constituents as they cycled through the CPB circuit (9).

Several conducted randomised controlled trials, including Octopus, PRAGUE-4 and Randomized On/Off Bypass, showed no significant difference in the early (30-day) composite outcome of complications or death (10). However, studies have suggested some benefit of OPCAB in high-risk patients. A recent meta-analysis of randomised control trials demonstrated a significant linear correlation (*P* < 0.01) between the patient risk profile and reduced perioperative morbidities (myocardial infarction and stroke) besides all-cause mortality (11).

Cannulation or cross-clamping of the aorta in patients with extensive disease of the ascending aorta carries a significant risk of stroke. Hence, OPCAB is the preferred approach by some surgeons who are familiar with its technical nuances (12). This is further supported by recently published reports, which emphasised that the most important strategy on reducing the risk of neurological complications is by avoiding aortic manipulation (13, 14).

### OPCAB: Pearls and Pitfalls

**Figure 1:**
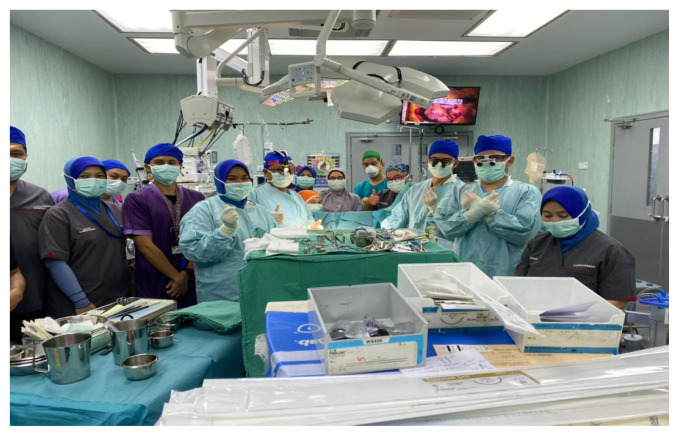
USM and UiTM team which involved the cardiac surgeons, cardiac anaesthesiologists, perfusionists and other supporting staffs

Conducting a pre-operative team briefing prior to cardiac surgery has been noted to improve communication and reduce errors and costs, according to a pilot study performed at Mayo Clinic (15). A 5-min briefing can be conducted in the vicinity of or in the operating room prior to the first surgical case of the day. Each team member will discuss his or her role in the procedure and convey any concerns specific to the surgery or the patient (Figure 1). Our patient has a normal body habitus with good effort tolerance, metabolic equivalents (METS > 4) and no failure symptoms. Affected vessels were left anterior descending (LAD) with long chronic total occlusion from proximal to distal, left circumflex (LCX) with 50% stenosis and diffused disease of the right coronary artery (RCA), with collateral distribution from the LCX. Left ventricular ejection fraction was 61%.

In addition to the standard and essential haemodynamic monitoring, a cerebral oximeter was used to ensure adequate cerebral oxygenation. Transesophageal echocardiography (TOE) is a key monitoring tool in modern cardiac surgery. Being competent in TOE is an added value in managing the haemodynamics during OPCAB. It provides continuous real-time monitoring of the overall cardiac function (16), facilitates goal-directed fluid therapy and provides valuable insight on haemodynamic derivations such as cardiac output and stroke volume (17).

**Figure 2:**
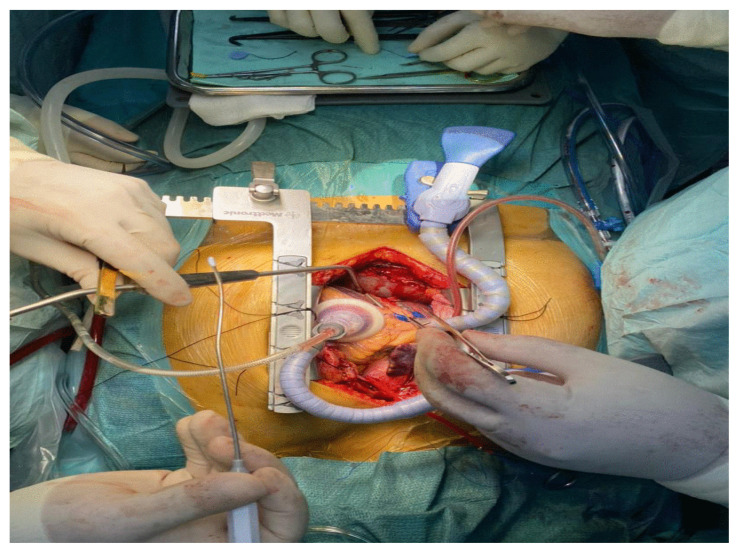
Placement of the heart positioner and stabiliser after cardiac manipulation for distal anastomosis of the graft

Cardiac positioning is determined as a fundamental step for the surgeons to reach their targets during OPCAB. The lifting and rotating of the heart will lead to frequent haemodynamic perturbations. Thus, the cardiac anaesthesiologist should be well-versed with the surgeon’s OPCAB approach, so that each cardiac manipulation can be anticipated and managed promptly. After the manipulation, the position was maintained by a heart positioner (Starfish^TM^) and further stabilised with Octopus^TM^ and Genzyme Immobilizer^®^ (Figure 2). Intraluminal shunts were placed within the anastomotic vessel, allowing distal perfusion during bypass grafting. Left internal mammary artery was anastomosed at the middle segment of the LAD. Saphenous vein graft was harvested from the right leg and anastomosed at the middle segment of the right posterolateral branch of RCA. In total, there were two distal and one proximal anastomoses performed.

Maintenance of a low normal heart rate ranging between 55 beats and 70 beats per min can improve the myocardial oxygen balance by decreasing the myocardial oxygen consumption besides providing an ideal surgical field for the surgeon (18, 19). The sympatholytic and vagomimetic properties of remifentanil provides a favourable condition for myocardial protection during OPCAB (18). Remifentanil has a predictable pharmacokinetic profile and rapidly achieves a steady-state plasma level. When compared with fentanyl or sufentanil, remifentanil has been shown to reduce the time of mechanical ventilation, cardiac troponin release, and length of hospital stay in inpatients undergoing surgical coronary revascularisation (20).

Prior to positioning for distal anastomoses, the primary aim is to achieve adequate preload; mean arterial pressure (MAP) is further maintained with vasopressor infusion. Greater than normal filling pressures were required to maintain ventricular filling during anastomoses of posterior and lateral targets. Most of the time, the speed of heart positioning is more dramatic than the displacement itself. Progressive heart positioning, timely MAP adjustments of more than 60 mmHg with intermittent boluses of vasopressor and Trendelenburg position may minimise on the haemodynamic disturbances. After completing all distal anastomoses, the systolic blood pressure was aimed between 90 mmHg and 100 mmHg prior to application of side-biting clamp for proximal anastomoses at the ascending aorta.

Close collaboration and good communication between the surgeons and anesthesiologists are pivotal in determining the success of the surgery. It is crucial for the surgeons to pre-emptively communicate with the anaesthesiologist prior to heart manipulation or positioning for prompt acute management of the haemodynamics. Simple maneuver such as small positional change or interventions such as intracoronary shunt or atrial pacing may significantly improve the ability to maintain stability. During anastomoses, significant haemodynamic disturbances such as severe hypotension, extreme bradycardia or recurrent dysrhythmias might occur. It may be necessary to advise the surgeon to stop the anastomosis and let the heart down, allowing it to re-fill and recover. This is at the expense of the coronary artery being still occluded and opened. In the event when both surgeon and anaesthesiologist are concerned of impending cardiovascular arrest before finishing the anastomoses, conversion to on-CPB should be initiated. Decision to convert to either a pump-assisted beating heart (also known as on-pump beating heart) or conventional CABG (paralysed heart) needs to be individualised from case-to-case basis. A good surgeon-anaesthesiologist combo should be able to give ample time to the perfusionist team to prepare the CPB machine before the haemodynamic deteriorates and the heart arrests. Consensual decision for conversion to CPB is a matter of safety and should not be viewed as a failure when performing OPCAB.

## Conclusion

Patient selection, meticulous attention to detail and acquisition of new sets of skills in terms of heart positioning and haemodynamic management are fundamental in ensuring a successful OPCAB due to its narrow margin of safety compared to conventional CABG. Leaving our comfort zone and a significant paradigm shift from routine conventional CABG to OPCAB can be challenging, but this introduces us to a different level of flexibility in offering the best possible and perhaps a safer option for certain group of patients.
